# Medical students’ empathy and attitudes towards professionalism: Relationship with personality, specialty preference and medical programme

**DOI:** 10.1371/journal.pone.0215675

**Published:** 2019-05-02

**Authors:** Colm M. P. O’Tuathaigh, Alia Nadhirah Idris, Eileen Duggan, Patricio Costa, Manuel João Costa

**Affiliations:** 1 Medical Education Unit, School of Medicine, University College Cork, Cork, Ireland; 2 School of Medicine, University College Cork, Cork, Ireland; 3 Life and Health Sciences Research Institute, School of Health Sciences, University of Minho, Braga, Portugal; Florida International University, UNITED STATES

## Abstract

**Background:**

Existing research has suggested that self-reported empathy in medical students is moderated by personality traits and diverse demographic and educational factors including age, gender, nationality, career aspirations, as well as year of curriculum. It is unclear how empathy, personality, and background factors might impact on students’ attitudes towards professionalism in medicine.

**Methods:**

A cross-sectional questionnaire-based study was conducted in first and final year medical students at an Irish medical school. The following instruments were administered: (a) Jefferson Scale of Empathy; (b) NEO Five-Factor Inventory (NEO-FFI-3); (c) Attitudes towards Professionalism Scale. Demographic and educational variables were also measured. Descriptive and correlational analysis was conducted to examine the association between empathy, personality, professionalism-related attitudes and additional measures. Regression analysis was used to examine determinants of attitudes towards professional behaviour.

**Results:**

Both selected NEO-FFI personality traits and empathy were independently associated with distinct categories of professional behaviour. Specifically, Openness to Experience was associated with higher empathy scores, and higher ‘Social responsibility’. Extraversion was linked with higher scores on the “Personal characteristics” and “Interactions with team” categories, while Conscientiousness was also positively associated with “Personal characteristics”. In agreement with previous studies, the personality traits most associated empathy were Agreeableness and Openness to Experience. Empathy did not vary according to programme year or career specialty preference.

**Conclusions:**

This study is the first to show that empathy and personality factors may act as determinants of students’ attitudes towards medical professionalism in a manner which is dependent upon category of professional behaviour.

## Introduction

Empathy is regarded as an essential physician attribute and is best defined a multidimensional construct, involving cognitive (the ability to understand and reflect someone else’s perspective), affective (ability to perceive subjectively another person’s inner experiences and natural feelings) and behavioural (the ability to competently communicate that one understands these feelings and perspectives) components [1–5].

Greater empathy is an important element in the development of a trustful doctor-patient relationship and has been positively correlated with optimal doctor-patient communication, more accurate diagnosis, and better treatment adherence [6–8]. However, studies linking physician empathy with patient care outcomes have largely relied on physician’s self-reported empathy levels and the validity of this approach has been questioned by an observed lack of correlation between patients’ measures of physician empathy and physician’s self-assessed empathy [9, 10]. In medical students, self-reported empathy was positively associated with increased communication skills scores in an objective clinical skills examination (OSCE) [11].

Hojat and colleagues note that the Jefferson Scale of Empathy (JSE), one of several psychometric measures used to measure physician empathy, was developed to measure a predominantly cognitive (rather than an affective or emotional) form of empathy [12]; results of factor analyses on the JSE have revealed the following three factors: “perspective taking”, “compassionate care’ (understanding of patients’ emotions and experiences), and “standing in the patients’ shoes’ (ability to see things from the patients’ perspective) [13, 14].

Some authors have suggested that there is an erosion in empathy during medical school, specifically, as students progress through the clinical years [14, 15]. However, studies which have investigated this phenomenon have demonstrated contradictory results, and a recent scoping review highlighted that the results regarding a decline in empathy were largely inconclusive, principally due to variability in terms of design and instrumentation, and that changes may be minimal or non-existent [5].

Empathy is typically included in conceptualisations of medical professionalism [16] Accrediting bodies worldwide, including the General Medical Council UK [16] and Association of American Medical Colleges (AAMC; [17]) have incorporated the development of empathy into guidelines and recommendations for fostering professionalism both undergraduate and postgraduate medical curricula. Although few studies have specifically focused on the relationship between self-reported empathy and the development of professional attitudes and attributes, studies have demonstrated that empathy development of medical students is associated with professionalism in clinical practice [18, 19]. Against the backdrop of data showing that doctors disciplined by regulatory bodies often demonstrate unprofessional attitudes and behaviours during their undergraduate education [20, 21], it is important to identify how attributes such as empathy which might influence the development of attitudes towards professionalism in clinical practice.

Empathy in medical students has also been examined in relation to the influential Big Five personality model (NEO-FFI; [22]), which has focused on the following five traits: Openness to Experience (O), Conscientiousness (C), Extraversion (E), Agreeableness (A), and Neuroticism (N). NEO-FFI inventory has been used to confirm a relationship between selected personality traits (principally A, E, C, and O) and different empathy measures; specifically, higher scores for both the O and A traits were able to identify the more empathic students [23, 24]. In contrast, a recent study of Japanese medical students revealed no association between JSE scores and O, N or C, while only A was weakly correlated with JSE [25]; these authors argued that personality measures are subject to cultural influences. Personality traits, as defined in the five-factor model, have also been independently investigated in relation to faculty ratings of resident performance across 12 orthopaedic surgery programs across the United States [26]. These authors reported that A was consistently associated with multiple performance measures including ratings of professionalism, and communication and interpersonal skills ratings.

To date, no study has explicitly examined the relative influence of personality traits and attributes such as empathy towards attitudes towards professionalism in a medical student population. This study was designed to explore the putative association between personality, psychometrically measured empathy, and attitudes towards medical professionalism in medical students as measured by a validated tool which focuses on four aspects of medical professionalism (personal characteristics; interaction with patients; social responsibility; interactions with the health). We also sought to investigate further the association between empathy and personality (as measured in the NEO-FFI) in an Irish medical school sample. Lastly, we sought to determine and compare empathy scores according to gender, year of study, mode of entry (direct-entry, graduate-entry), and career specialty preferences.

## Materials and methods

### Design

Undergraduate medical students (n = 241) in the classes of 2016 and 2017 at an Irish medical school (University College Cork) participated in this cross-sectional study. Study participants were enrolled in either the direct-entry 5-year undergraduate programme (years 1 and 5) or the graduate-entry 4-year medicine programme (years 1 and 4). Students completing other undergraduate programme years, or registered on alternative degree programmes were excluded from participation in this study. A convenience sampling method was employed. A multi-mode method of survey distribution was employed, with students either completing a paper survey at the end of a lecture or completing a web-based survey distributed to their email address. Participation was made voluntary and confidentiality was guaranteed. The study was approved by the Clinical Research Ethics Committee of the Cork Teaching Hospitals. The survey was anonymous and responses were not linked with the participants' identity, and the Clinical Research Ethics Committee consequently provided a waiver of the requirement for signed consent.

### Instruments

Participants were administered a questionnaire battery consisting of the following items and instruments:

(a) Demographic and educational background: age, gender, nationality, medical programme (direct-entry vs graduate-entry), year of programme (first vs final year). (b) Choice of speciality as a future career was based on relative ranking of the following four different specialty categories: (1) diagnostic: perform diagnostic or specialized technical procedures e.g. radiology, pathology, haematology, anaesthesiology; (2) therapeutic: make technical or specialized procedures requiring motor skills e.g. orthopaedic surgery, neurosurgery, ophthalmology, urology; (3) internal medicine: provide episodic or long-term care, dealing with a specific set of medical problems which may include instrumentation and technical interventions e.g. cardiology, psychiatry, dermatology, obstetrics and gynaecology; (4) primary care: provide initial health assessment of disease, preventative education, intervention and comprehensive care to a variety of medical problems e.g. general practice, paediatrics.

(c) Jefferson Scale of Empathy (JSE)–student version—that includes 20 items answered on a Likert type scale: from 1 (strongly disagree) to 7 (strongly agree), and aggregated in three factors: “Perspective Taking” (10 items), “Compassionate Care” (8 items), and “Standing in the Patient's Shoes” (2 items)

(d) The five personality dimensions, Neuroticism, Extraversion, Agreeableness, Openness to Experience and Conscientiousness, were measured with the NEO-FFI inventory [22]. It uses a 5-point Likert scale ranging from 0 (strongly disagree) to 4 (strongly agree) and includes 60 items. Subscale scores are calculated for each dimension by summing the item scores that correspond to the related subscale.

(e) Attitudes towards professionalism were measured using a questionnaire developed and described previously [27, 28]. This survey tool grouped professionalism under the following four categories: ‘Personal characteristics’; ‘Interaction with patients’; ‘Social responsibility’; ‘Interactions with the health care team’ (Table 1). Additionally, several items are included to examine ‘Strategies for developing professionalism’ (data not included in present analysis). Participants were asked to rate the items listed above on an ascending 5-point Likert scale with rating options: Not at all important, somewhat important, neutral, important and very important. The average time needed to complete the questionnaire battery was 15 minutes. Missing data was handled for instruments (c)-(e) by replacing the missing item with the average of the other items in the scale. For items under (a) and (b), where responses were missing, relevant analyses were restricted to participants who had completed those items.

**Table 1:** Title and item breakdown for four categories of professional behaviours [27, 28].

Category Title	Category Items
**Personal Characteristics**	Internal Motivation
	Punctuality
	Attendance
	Appearance
	Reliability in Patient Care
	Commitment to Learning
	Knowledge of Limits
	Response to Assessment
	Self-improvement
	Honesty
	Avoiding Abuse of Power
	Adhering to Ethics
	Accountability for Decisions
**Interactions with Patients**	Respect for Patients
	Patients’ Involvement in Decisions
	Confidentiality
	Respect for Family
	Patient Concerns First
**Social Responsibility**	Treating the Underprivileged
	Improve Access to Care
	Just Distribution of Resources
	Teach and Disseminate Knowledge
	Advocate for Patients
	Manage Conflicts of Interest
**Interaction with Team**	Respect Other Members
	Report Dishonesty

### Data analysis

All the data was entered and analysed using SPSS v.24. Descriptive statistics were used to summarise student demographic and educational background data, as well as indices of central tendency and dispersion for item or summary scale scores across the various study instruments. Cronbach's alpha was used to assess internal consistency of categories of professional behaviour. Independent t-tests or Mann-Whitney U tests (for ordinal and non-parametric data) were used to assess changes in total JSE scores, NEO-FFI total dimension scores, and total professionalism category scores based on gender, year of study (1^st^ vs final year), medical programme (direct-entry vs graduate-entry). Pearson’s r correlation test was used to measure the strength of association between total JSE, total NEO-FFI dimension scores, and total professionalism category scores. Multiple linear regression analysis was used to identify significant predictors of professionalism category score variation in the present sample.

## Results

### Demographics

A total of 241 students participated in this study, providing a response rate of 59.1% (241/408). Of this sample, 49.2% (n = 117) were female, and the total sample was distributed across the following age (years) categories: 18–22 (46.6%), 23–27 (43.3%), 28–32 (8.8%), 33–37 (0.8%), 38–42 (0.5%). The total breakdown of the student sample across years 1 or 4/5 of the direct-entry or graduate-entry programme is as follows: direct-entry year 1: n = 102, 42.3%; direct-entry year 5: n = 72, 29.9%; graduate-entry year 1: n = 41, 17.0%; graduate-entry year 4: n = 26, 10.8%. The majority of students classified their region as Christianity (50.8%), followed by Islam (12.6%) and no religion (27.7%), and students’ nationalities were as follows: Irish (58.1%); Malaysian (15.4%); Canada (9.5%); EU (including UK; 2.9%); other (14.1%). No significant differences were observed between demographic characteristics of our study sample (n = 241), and the total eligible study population (n = 408; S1 Table), aside from medical programme, where graduate-entrants were under-represented in the study sample (*X*^2^ = 8.47, P<0.01).

### NEO-FFI and empathy

Statistically significant and positive correlations were observed between total JSE score and Openness to Experience (r  =  0.27, P = 0.01) and Agreeableness (r  =  0.15, P = 0.01). Table 2 provides a summary of descriptive statistics and correlational analyses. The magnitudes of correlations between personality dimensions and JSE scores were low to moderate, ranging from 0.07 to 0.31 for Conscientiousness and Openness to Experience respectively.

**Table 2:** Descriptive statistics (mean value ± standard error (SE)) and Correlations (Pearson’s r) between total JSE score and NEO-FFI personality traits.

	Total JSE	Extraversion	Neuroticism	Conscientiousness	Openness to Experience	Agreeableness
Mean SE	109.90 0.96	43.46 0.44	32.75 0.60	43.76 0.52	41.91 0.42	44.96 0.43
Total JSE	-	0.09	0.07	0.07	0.27 **	0.15 *
Extraversion	-	-	-0.45 **	0.28	0.08	0.35 **
Neuroticism	-	-	-	-0.31 **	0.08	-0.21 **
Conscientiousness	-	-	-	-	-0.06	0.20 **
Openness to Experience	-	-	-	-	-	0.14 *
Agreeableness	-	-	-	-	-	-

* P < 0.05

** P < 0.001.

### Attitudes to professionalism

Cronbach’s alpha (α) analysis of the results of the present study demonstrated that each of the attitudes to professionalism categories, bar the ‘Interactions with team’ category possessed high internal consistency: ‘Personal characteristics’ (α = 0.87); ‘Interactions with patients’ (α = 0.76); ‘Social responsibility’ (α = 0.82); ‘Interactions with team’ (α = 0.42); ‘Strategies for developing professionalism’ (α = 0.69). Self-reported empathy was positively correlated with higher scores on the ‘Personal characteristics’ (r = 0.21, P = 0.01) and ‘Interactions with patients’ domains (r = 0.19, P = 0.01). Extraversion was significantly and positively correlated with ‘Personal characteristics’ (r = 0.33, P = 0.01), ‘Interactions with patients (r = 0.22, P = 0.01), ‘Social responsibility’ (r = 0.21, P = 0.01), and ‘Interaction with team members’ (r = 0.27, P = 0.01). Agreeableness was also positively correlated with ‘Personal characteristics’ (r = 0.23, P = 0.01), ‘interactions with patients (r = 0.19, p = 0.012), and ‘interaction with team members’ (r = 0.22, P = 0.01). Low Neuroticism was associated with increased ‘Social responsibility’ (r = 0.22, P = 0.01). Conscientiousness was positively correlated with ‘Personal characteristics’ (r = 0.29, P = 0.01) and ‘Interactions with patients’ (r = 0.19, P = 0.01). The strength of correlations between personality dimensions and professional behaviour category scores were predominantly weak.

### Career specialty preference

The most popular career specialty categories, in descending order, were as follows: internal medicine (mean rating = 2.12, SE = 0.06); primary care (mean rating = 2.37, SE = 0.07); therapeutic (mean rating = 2.51, SE = 0.08); diagnostic (mean rating = 2.94, SE = 0.07). Specialty preference was not associated with self-assessed empathy, but students selecting an internal medicine specialty demonstrated significantly higher neuroticism scores relative to those who selected primary care (F (3, 199) = 3.42, P = 0.02). Additionally, Agreeableness scores were higher among students selecting primary care relative to those selecting a therapeutic specialty (F (3, 199) = 6.25, P < 0.001). No significant association was observed between career specialty preference and professionalism category scores (all P > 0.05).

### Gender

No gender-related differences were observed for JSE scores (t(215) = 0.60, P = 0.55) or any of the NEO-FFI dimensions (Neuroticism, t (202) = 0.06, P = 0.86; Extraversion, t (202) = 0.94, P = 0.65; Openness to Experience, t (202) = 0.81, P = 0.07; Agreeableness, t (202) = 0.79, P = 0.43; Conscientiousness, t (202) = 1.69, P = 0.09). Females displayed significantly higher importance ratings than males for items belonging to the ‘Social responsibility’ professionalism-related category (t(182) = 3.10, P = 0.01). No gender differences were observed for the remaining three categories of professional behaviour, nor were any differences observed between males and females in relation to specialty preference (all P > 0.05).

### Year of study

No significant differences between students in the first years vs final year of study in relation to empathy scores (t(217) = 1.14, P = 0.25) or three of the four NEO-FFI dimensions (Extraversion, t (204) = 0.88, P = 0.38; Openness to Experience, t (204) = 0.72, P = 0.47; Agreeableness, t (204) = 0.33, P = 0.74; Conscientiousness, t (204) = 0.75, P = 0.46). Final year medical students displayed significantly higher neuroticism relative to first year students (t (204) = 2.19, P = 0.03). A reduction in importance ratings for the ‘Social responsibility’ professionalism category was observed for final year vs first year students (t (183) = 2.93, P < 0.01). In relation to specialty preference, first year students displayed higher preference for the ‘diagnostic’ (U = 4969, z = 2.06, P = 0.04) and ‘internal medicine’ specialties (U = 5189, z = 2.38, P = 0.02), while the converse pattern was observed for ‘therapeutic’ specialties (U = 4538, z = 3.98, P = 0.01).

### Medical programme

Graduate-entry students showed increased JSE values relative to direct-entry (t (217) = 2.07, P = 0.04), as well as increased Openness to Experience (t (204) = 3.80, P = 0.01) and Conscientiousness (t (204) = 2.10, P = 0.04). Direct-entry students provided higher importance ratings relative to graduate-entry students for ‘Social responsibility’ professionalism items (t (183) = 2.20, P = 0.03). No other significant differences related to medical programme type were observed.

### Multiple linear regression

To determine the extent to which self-reported empathy, personality dimensions and selected demographic/educational variables influence the professionalism-related category scores, linear regression analyses were conducted. Table 3 provides a summary of the factors affecting professionalism category score variation in the present sample. Extraversion and Conscientiousness traits emerged as significant positive predictors of scores in the ‘Personal Characteristics’ category (P < 0.05). Both empathy scale scores and low neuroticism were associated with improved scores in the ‘Interactions with patients’ category (P < 0.05). Openness to Experience scores were associated with better scores under the ‘social responsibility’ category (P = 0.03), while extraversion positively predicted increased scores under the “Interaction with team” category (P = 0.01).

**Table 3:** Results of multiple linear regression modelling for each of the four professionalism categories (‘Personal characteristics’; ‘Interactions with patients’; ‘Social responsibility’; ‘Interactions with team’). B denotes the unstandardised variable estimate. SE denotes the standard error of the variable estimate. Beta denotes the standardised variable estimate. The t statistic is the coefficient divided by its standard error.

	Dependent Variable: Personal Characteristics					Dependent Variable: Interactions with Patients					Dependent Variable: Social Responsibility					Dependent Variable: Interactions with Team				
Model	Unstandardised Coefficients		Standardised Coefficients			Unstandardised Coefficients		Standardised Coefficients			Unstandardised Coefficients		Standardised Coefficients			Unstandardised Coefficients		Standardised Coefficients		
	B	Std. Error	Beta	t	Sig.	B	Std. Error	Beta	t	Sig.	B	Std. Error	Beta	t	Sig.	B	Std. Error	Beta	t	Sig.
[Constant]	2.01	0.54		3.73	0.00 **	3.19	0.61		5.26	0.00 **	1.54	0.66		2.35	0.02 *	2.29	0.63		3.66	0.00 **
Gender	0.05	0.07	0.06	0.66	0.51	-0.05	0.08	-0.05	-0.54	0.59	0.04	0.09	0.04	0.45	0.65	0.05	0.09	0.05	0.54	0.59
Age Range	0.07	0.06	0.12	1.13	0.26	0.05	0.06	0.08	0.80	0.43	0.14	0.07	0.19	1.95	0.05	0.09	0.07	0.14	1.37	0.17
Medical Programme	0.08	0.11	0.07	0.72	0.47	0.12	0.12	0.11	1.02	0.31	0.17	0.13	0.14	1.32	0.19	0.03	0.12	0.03	0.28	0.78
Empathy [JSE]	0.004	0.002	0.13	1.55	0.13	0.01	0.003	0.21	2.40	0.02 *	0.01	0.003	0.15	1.73	0.09	0.002	0.003	0.07	0.76	0.45
Neuroticism	0.003	0.01	0.07	0.68	0.50	-0.01	0.01	-0.21	-2.02	0.05 *	0.00	0.01	0.01	0.05	0.96	0.01	0.01	0.11	1.06	0.29
Extraversion	0.02	0.01	0.32	3.23	0.002 **	0.01	0.01	0.09	0.87	0.38	0.02	0.01	0.18	1.80	0.08	0.02	0.01	0.27	2.58	0.01 *
Openness	0.01	0.01	0.14	1.52	0.13	0.01	0.01	0.08	0.79	0.43	0.02	0.01	0.21	2.20	0.03 *	0.003	0.01	0.03	0.34	0.74
Agreeableness	-0.003	0.01	-0.04	-0.39	0.70	-0.004	0.01	-0.05	-0.50	0.62	0.002	0.01	0.03	0.26	0.80	0.01	0.01	0.13	1.26	0.21
Conscientiousness	0.01	0.01	0.20	2.27	0.03 *	0.01	0.01	0.15	1.60	0.11	0.01	0.01	0.14	1.48	0.14	0.004	0.01	0.05	0.58	0.57

* P < 0.05

** P < 0.001.

## Discussion

In the present study, several of the NEO-FFI personality traits were independently associated with distinct categories of professional behaviour in the current sample of medical students. Based on the multiple regression analyses, Openness to Experience was associated with higher empathy scores, and higher ‘Social responsibility’ category scores; this latter category is focused on patient advocacy, improving access to care, and managing conflicts of interest. Agreeableness was also weakly correlated with increased JSE scores. Extraversion was linked with higher scores on the “Personal characteristics” and “Interactions with team” categories, while Conscientiousness was also positively associated with scores under the “Personal characteristics” category. Empathy levels also emerged as a significant positive predictor of score variation in the “Social responsibility professional behaviour category.

The most consistent personality traits associated with self-assessed empathy in the present sample were Agreeableness and Openness to Experience and this result resonates with previous studies [24]. Employing the Empathy Quotient (EQ) and Interpersonal Reactivity Index (IRI) indices of empathy, Melchers et al [29] identified agreeableness (and conscientiousness) as the most important predictor of affective and cognitive empathy (measured by the respective IRI subscales) in an international sample of university students as well as for a one-dimensional empathy score (measured by the EQ). Similarly, in a sample of Romanian health professions students, empathy (as measured using the empathy quotient (EQ) scale) was positively associated with Agreeableness [30]. Both Agreeableness and Openness to Experience have previously been linked with superior academic and clinical performance in studies of medical students and physicians [31, 32]. Specifically, Openness to Experience has been linked with increased cognitive flexibility [33], as well as empathy measures which would be expected to prove beneficial in academic or clinical work measures [34]. Additionally, high Extraversion has previously been shown to be beneficial for physician’ collaboration and communication skills in professional practice [34], and this is consistent with the finding that students scoring high for Extraversion also provide high scores under the “Interactions with team” professionalism category.

Conscientiousness is a trait that he has previously been linked with medical professionalism, including self-discipline and attention to detail [35]. Studies employing tools have been developed to specifically measure this aspect of professionalism (e.g. the ‘Conscientiousness Index’) show significant correlations with individual estimation of student professionalism as based on both student peer [36] and faculty ratings [37]. Similarly, occurrence and frequency of behaviours associated with conscientiousness, including course evaluation compliance during the preclinical years, has been associated with evidence for professionalism in the clinical setting [38]. Not surprisingly therefore, the “Personal characteristics” professionalism category, which included items like punctuality, attendance, and reliability in patient care, was associated with Conscientiousness in the present study.

Our findings showed statistically significant associations between dimensions of empathy and personality, and different facets of medical professionalism. However, the magnitude of their contribution to variation across these attitudinal measures was limited. Medical professionalism has been conceptualised as a multifaceted, complex, and dynamic concept [39]. A review of physician-related and environmental influences on individual physicians’ professionalism, suggests a complex and interactive multifactorial model which includes personal attributes (e.g. personality traits, interpersonal skills) and other individual factors (e.g. career motivation); physician well-being, which is greatly affected by profession-specific stressors; and environmental factors, including institutional features and practice characteristics [40]. In that context, this work better characterises one or more nodes in this interactive network, providing new insight into the relative involvement of specific individual factors, empathy and personality, to expression of attitudes towards specific domains of medical professionalism. We report that self-reported empathy and personality measures are weak but significant predictors of attitudes to professional behaviour among medical students. Additionally, some personality characteristics were more closely related to specific domains of professionalism than others. Future research will be needed to investigate how personality and empathy contributions to professionalism, both shape or modify, or are modified, by other salient features including the stresses of medical education and qualities of the learning environment. Our findings of low correlations with empathy and professionalism measures is not aligned with the concept that empathy is part of the professional conduct of a doctor. In our view, such low correlations may originate from the fact that the JSE scores represent only a share and not the whole dimensions of the participants’ empathy, as we discuss below.

Medical students’ empathy considering the stage in training, gender and future career specialty preference was investigated in the present study. Even though final year students exhibited higher mean empathy values, the results of our cross-sectional analysis showed no significant difference between the first and final year students. These data are congruent with those reported by previous studies carried out in other parts of the world [41, 42]. This higher mean score observed for final year students has been observed in other cross-sectional investigations of changes of empathy during medical school; a different profile has been observed for longitudinal studies, where a mixed or empathy decline result has been reported [5]. Additionally, we observed an absence of significant gender differences with regards to the JSE scores; this is inconsistent with the oft-reported finding from Western studies that female students are more empathic than males [43, 44]. It is, however, harmonious with a few studies conducted in Asian context [45, 46]. It is interesting to note that in this study, almost one fourth of the participants are of Asian nationalities which might have contributed to this failure to observe any change or decline.

Specialty preference was not associated with self-assessed empathy in the current analysis. Other studies have also found that desired specialty is not significant in determining the more empathic students (e.g. [47, 48]). Additionally, research which has looked at motivation to study medicine has revealed a relatively weak association between person-orientation and JSE values [18, 49], and selection/admission tests which are designed to assess non-academic attributes have been shown to have limited association with empathy during medical school [42]. In contrast, some studies have shown that medical students who indicate a high preference for people-oriented specialties score higher on the JSE [6, 49]. Based on standardised measures of patient experience, one large-scale (n = 847) US study demonstrated that physician across four specialties (obstetrics-gynaecology, paediatrics, psychiatry, and thoracic surgery) were associated with higher empathy values [50]. Specialty preference was also not associated with variation in professionalism category scores.

The present study showed that some personality dimensions were related to the choice of a given medical specialty. Specifically, Neuroticism was higher among students selecting an ‘Internal medicine’ specialty relative to those who selected ‘primary care’, while Agreeableness scores were higher among students selecting ‘primary care’ relative to those selecting a ‘therapeutic’ specialty. Our results were in accordance with the prospective assessment by Maron et al. [51], which reported high neuroticism in students selecting internal medicine. Consistent with these data, a recent study involving a sample of Finnish physicians (N  =  2837) demonstrated that higher agreeableness was observed in physicians working in the private sector, and working in general practice or occupational health specialties [52]. Lower Agreeableness and Neuroticism was reported in physicians specialising in surgery. In contrast with the present study, the personality traits which was most consistently associated with career choice (including sector, amount of patient contact, preferred specialty) was Openness to Experience. Other studies have failed to demonstrate any association between five factor personality profiles and specialty preferences [53].

With respect to study limitations, the response rate to this research was stratified based on programme year, relatively lower among final year students due to outside clinical rotation and research commitments. The cross-sectional nature of this study also limits the ability to evaluate changes in the empathy or other aspects of professionalism in the students which would be better in a longitudinal cohort study. This study is also a single institutional study and cannot be generalised into all Irish students, given only 53% of the participants in this school are Irish students. The usage of self-reported questionnaire to measure multifaceted concepts such as professionalism and, arguably, empathy also limits the validity of the data produced. Some authors have documented that actual professional behaviour is not always in agreement with self-reported attitudes towards professionalism in practicing physicians [54] and a study in Brazil found that patient ratings of doctors’ empathy did not correlate with self- assessments [10]. Opposite findings originated from a recent international validation study employing a using a novel professionalism measurement tool for physicians and nurses, demonstrated a significant association between self-reported positive attitudes towards professionalism and participation in professionalism-linked behaviours including quality improvement initiatives [55]. Additionally, recent studies have identified significant correlations between self-reported empathy and quality of patient-centred communication in medical trainees [56], trainee dentists [57], and emergence medicine physicians [58]. The challenge of clarifying and characterizing the specific share of clinical empathy or professionalism captured by self-reported measures calls for further research. In the meanwhile, self-reported measures remain feasible for faculty, resident supervisors and program evaluators to obtain indicators related to some of the complex dimensions of these constructs.

## Conclusions

Our study is the first examination of how personality and self-reported empathy impact upon attitudes towards different categories of professional behaviour in medicine. Consistent with previous results, it identified Agreeableness and Openness to Experience as correlates of empathy, while both Extraversion, Openness to Experience and Conscientiousness were significant predictors of perceived importance ascribed to different aspects of medical professionalism. Neither year of study, age, nor career specialty preference had any effect on empathy values, but graduate-entry medical students demonstrated increased empathy and both increased Openness to Experience and Conscientiousness relative to direct-entry students.

## Supporting information

S1 Table Comparison of study participants’ and total eligible sample demographic characteristics. (DOCX)
