# Supplementary material for: Medical students’ empathy and attitudes towards professionalism: Relationship with personality, specialty preference and medical programme
Source: PLoS One. 2019 May 2;14(5):e0215675. doi: 10.1371/journal.pone.0215675 (PMC6497245; doi:10.1371/journal.pone.0215675)
Supplement: S1 Table — (DOCX) [file pone.0215675.s001.docx]

| **Variable** | **Study sample (n=241)** | **Total study population (n=408)** |
| --- | --- | --- |
| Age (Y, %) |  |  |
| 18-22 | 46.6% | 32.4% |
| 23-27 | 43.3% | 56.8% |
| 28-32 | 8.8% | 9.3% |
| 33-37 | 0.8% | 1.2% |
| 38-42 | 0.5% | 0.3% |
| Gender , female (%) | 49.2% | 51.4% |
| Medical programme |  |  |
| Direct-entry | 83.6% | 65.7% |
| Graduate-entry | 16.4% | 34.3% |
| Nationality (%) |  |  |
| Irish | 58.1% | 69.1% |
| Non-Irish | 41.9% | 30.9% |

**S1 Table: Comparison of study participants’ and total eligible sample demographic characteristics**
